# Interaction Energy between Two Separated Charged Spheres Surrounded Inside and Outside by Electrolyte

**DOI:** 10.3390/membranes12100947

**Published:** 2022-09-28

**Authors:** István P. Sugár

**Affiliations:** Department of Neurology, Icahn School of Medicine at Mount Sinai, New York, NY 10029, USA; istvansugar0@gmail.com

**Keywords:** Debye length, screened potential, charge–charge interaction energy

## Abstract

By using the recently generalized version of Newton’s shell theorem, analytical equations are derived to calculate the electric interaction energy between two separated, charged spheres surrounded outside and inside by electrolyte. This electric interaction energy is calculated as a function of the electrolyte’s ion concentration, temperature, distance between the spheres and size of the spheres. At the same distance between the spheres, the absolute value of the interaction energy decreases with increasing electrolyte ion concentration and increases with increasing temperature. At zero electrolyte ion concentration, the derived analytical equation transforms into the Coulomb Equation Finally, the analytical equation is generalized to calculate the electric interaction energy of N separated, charged spheres surrounded by electrolyte.

## 1. Introduction 

Recently, an analytical equation was derived that is a generalization of Newton’s shell theorem [1,2]. By using this equation (see Equation (1)), one can calculate the electric potential, V, around a surface-charged sphere surrounded inside and outside by electrolyte at a distance *Z* from the center of the sphere (see also Equation (9) in e.g., [1]):

(1) $$V\left(Z,Q\right)=\frac{k_{e}Q\lambda_{D}}{\varepsilon_{r}ZR}e^{-\frac{Z}{\lambda_{D}}}\;sinh\left(\frac{R}{\lambda_{D}}\right)\;\mathrm{at}\;Z>R$$

where *k_e_* = (4π*ε*_0_)^−1^ is the Coulomb’s constant, $\varepsilon_{0}$ is the vacuum permittivity, *λ_D_* is the Debye length, *Q* is the total charge of the homogeneously charged surface of the sphere of radius *R* and *ε_r_* is the relative static permittivity of the electrolyte. Note that recently, by using Equation (1), the electric energies have been calculated [3], such as the potential electric energy needed to build up a surface-charged sphere, and the field and polarization energy of the electrolyte inside and around the surface-charged sphere. In this paper, Equation (1) is used to derive an analytical equation to calculate the interaction energy between two separated surface-charged spheres surrounded inside and outside by electrolyte. This equation is a generalization of Coulomb’s law [4] that gives the interaction energy between two charges embedded in a vacuum. By means of the equation derived in this paper, one may get closer to study the long-range charge–charge interaction between vesicles or cells. The head groups of membrane lipids have either a single charge (e.g., tetraether lipids [5,6]) or an electric dipole (e.g., phospholipids [7,8]). Theoretical models of lipid membranes usually focus on short-range (Van der Waals) lateral interactions between the nearest neighbor lipids and ignore the long-range charge–charge interactions [8]. This is because in the case of long-range interactions one has to consider the entire system rather than the lateral interactions between the nearest neighbor lipids only. It is much more difficult to model a lipid membrane containing single-charged head groups [9]. Between lipids with single-charged head groups there is long-range interaction, i.e., where the two-body potential decays algebraically at large distances with a power smaller than the spatial dimension [10], and, thus, when modeling this system one has to consider the entire system rather than the interactions between the nearest-neighbor lipids.

Deriving Equation (1), the general solution of the screened Poisson equation was utilized (see Equation (4) in [1] or (A5) in Appendix A), an equation that is valid if the electrolyte is electrically neutral [11]. It is important to note that the screened Poisson equation (Equation (A4)) is different from the Poisson—Boltzmann equation (see Equations (A1) and (A3)). The Poisson—Boltzmann equation can be used to calculate the potential energy of an arbitrary, electroneutral ion solution (i.e., electrolyte). However, for the solution (see Equation (A2)) one has to know the charge density of the ions in the electrolyte (i.e., the Boltzmann distribution; see Equation (A3)), which depends on the potential, *V*, itself. Thus, only an approximative solution is available (the Debye–Hückel approximation [12]), which is valid when |*z_i_qV/(k_B_T)*| ≪ 1 (where *q*: charge of a monovalent ion (either positive or negative), *z_i_*: charge number (or valence) of the *i*-th type of ion, *k_B_*: Boltzmann constant, *T*: absolute temperature). Using the screened Poisson equation (Equation (A4)), one can calculate the potential energy of an electrolyte that also contains external charges. The external charges are embedded into the electrolyte (like the charges of the surface-charged sphere) but are not part of the electrolyte itself. For the solution, one has to know the charge density of the external charges, $\rho_{ex}\left(\underline{r}\right)$ (see Equation (4) in [1] or Equation (A5) in Appendix A), i.e., distribution of the charges on the surface-charged sphere and not the distribution of the ions in the electrolyte. In our case, it is assumed that the charges on the surface of the sphere are homogeneously distributed and, in this case, Equation (1) is the exact solution of the screened Poisson Equation

Finally, we notice that by means of the analytical equation derived in this paper one can calculate the dependence of the electric interaction energy from the distance, charge and size of the spheres and from the electrolyte’s ion concentration and the temperature. In the case of our calculations, the surface-charge density of the charged spheres at every radius is *ρ*_*s*_ = −0.266 × *C*/m^2^. This is the charge density of PLFE (bipolar tetraether lipid with the polar lipid fraction E) vesicles if the cross-sectional area of a PLFE is 0.6 nm^2^ and the charge of a PLFE molecule is −1.6 × 10^−19^ *C* [5,6].

## 2. Model

**Figure 1:**
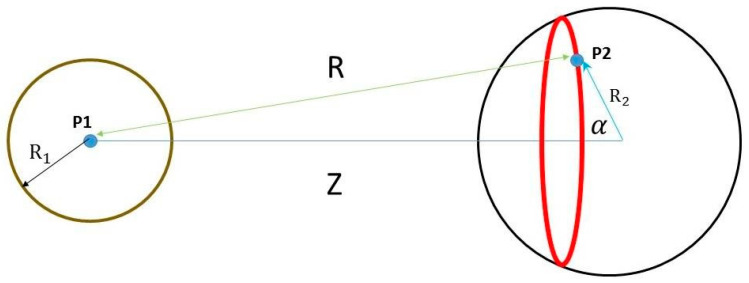
*Two charged spheres*: left circle represents a charged sphere of radius *R*_1_ and its total surface charge is *Q*_1_. Right circle represents a charged sphere of radius *R*_2_ and its total surface charge is *Q*_2_. The distance between the centers of the two spheres is *Z*. The potential created by the left charged sphere is calculated at point P2.

Figure 1 shows two charged spheres. The distance between the centers of the two spheres is *Z*. The potential created by the left charged sphere is calculated at point P2.

The red ring represents charges on the right charged sphere. Their distance from point P1 is *R*. α is the angle between vector *Z* and a vector pointing from the center of the right sphere to any of the points (P2) of the red ring.

Based on the generalized shell theorem [1], the electric potential created by the left charged sphere at point P2 is Equation (2):

(2) $$V\left(R\right)=\frac{k_{e}Q_{1}\lambda_{D}}{\varepsilon_{r}RR_{1}}e^{-\frac{R}{\lambda_{D}}}\;sinh\left(\frac{R_{1}}{\lambda_{D}}\right)$$

The distance between point P1 and any of the point charges located on the red ring is Equation (3):

(3) $$R\left(\alpha ,Z,R_{2}\right)=\sqrt{{\left(R_{2}sin\left(\alpha \right)\right)}^{2}+{\left(Z-R_{2}cos\left(\alpha \right)\right)}^{2}}=\sqrt{{R_{2}}^{2}+Z^{2}-2ZR_{2}cos\left(\alpha \right)}$$

The interaction energy between the left charged sphere and the charges of the red ring is Equation (4):

(4) $$E\left(\alpha \right)d\alpha =V\left(R\right)\rho_{2}2R_{2}sin\left(\alpha \right)\pi R_{2}\times d\alpha =\;\frac{k_{e}Q_{1}\lambda_{D}}{\varepsilon_{r}R\left(\alpha ,Z,R_{2}\right)R_{1}}e^{-\frac{R\left(\alpha ,Z,R_{2}\right)}{\lambda_{D}}}sinh\left(\frac{R_{1}}{\lambda_{D}}\right)\frac{Q_{2}}{2}sin\left(\alpha \right)\times d\alpha$$

where 2*R*_2_sin (α)π*R*_2_ × *dα* is the surface area of the red ring.

Finally, the interaction energy between the left and right sphere is Equation (5):

(5) $$E=\int_{0}^{\pi }E\left(\alpha \right)d\alpha =A\int_{0}^{\pi }\frac{sin\left(\alpha \right)}{\sqrt{R^{2}+Z^{2}-2ZR_{2}cos\left(\alpha \right)}}e^{-\sqrt{R^{2}+Z^{2}-2ZR_{2}cos\left(\alpha \right)}/\lambda_{D}}d\alpha$$

where $A=\frac{k_{e}Q_{1}\lambda_{D}}{\varepsilon_{r}R_{1}}sinh\left(\frac{R_{1}}{\lambda_{D}}\right)\frac{Q_{2}}{2}$

Let us do the following substitution in the integral: *u* = cos (α).

Thus, in Equation (5) sin(α) *dα* can be substituted by −*du* and we get Equation (6):

(6) $$=A\int_{-1}^{1}\frac{1}{\sqrt{{R_{2}}^{2}+Z^{2}-2ZR_{2}u}}e^{-\sqrt{{R_{2}}^{2}+Z^{2}-2ZR_{2}u}/\lambda_{D}}du$$

Finally, let us do this substitution in Equation (6): $w=-\sqrt{{R_{2}}^{2}+Z^{2}-2ZR_{2}u}/\lambda_{D}$ and thus

$dw=\frac{ZR_{2}}{\lambda_{D}\sqrt{{R_{2}}^{2}+Z^{2}-2ZR_{2}u}}du$ and we get Equation (7):

(7) $$E=\frac{A\lambda_{D}}{ZR_{2}}\int_{w\left(u=-1\right)}^{w\left(u=1\right)}e^{w}dw=\frac{A\lambda_{D}}{ZR_{2}}{\left[e^{w}\right]}_{w\left(u=-1\right)}^{w\left(u=1\right)}$$

where

(8) $$w\left(u=-1\right)=-\frac{\sqrt{{R_{2}}^{2}+Z^{2}+2ZR_{2}}}{\lambda_{D}}=-\frac{\left(Z+R_{2}\right)}{\lambda_{D}}$$

while in the case of *Z* > *R*_2_:

(9) $$w\left(u=1\right)=-\frac{\sqrt{{R_{2}}^{2}+Z^{2}-2ZR_{2}}}{\lambda_{D}}=-\frac{\sqrt{{\left(Z-R_{2}\right)}^{2}}}{\lambda_{D}}=-\frac{\left(Z-R_{2}\right)}{\lambda_{D}}$$

Thus, from Equations (7)–(9) we get Equation (10):

(10) $$E\left(Z\right)=\frac{A\lambda_{D}}{ZR_{2}}\left[e^{-\frac{Z-R_{2}}{\lambda_{D}}}-e^{-\frac{Z+R_{2}}{\lambda_{D}}}\right]=\frac{A\lambda_{D}}{ZR_{2}}e^{-\frac{Z}{\lambda_{D}}}\times 2sinh\left(\frac{R_{2}}{\lambda_{D}}\right)=\;\frac{k_{e}Q_{1}Q_{2}{\lambda_{D}}^{2}}{\varepsilon_{r}R_{1}R_{2}Z}sinh\left(\frac{R_{1}}{\lambda_{D}}\right)sinh\left(\frac{R_{2}}{\lambda_{D}}\right)e^{-Z/\lambda_{D}}$$

where $Z>R_{1}+R_{2}$.

## 3. Results

**Figure 2:**
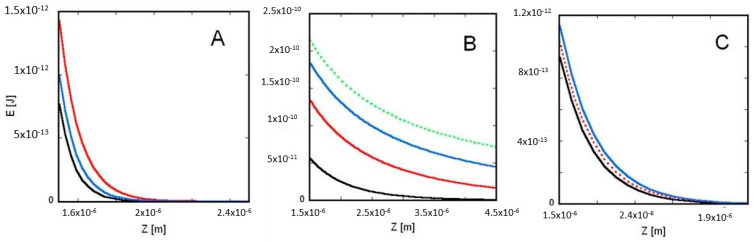
Interaction energy of two charged spheres surrounded by electrolyte (dependence from electrolyte’s ion concentration and temperature): the smaller sphere with radius *R*_1_ = 5 × 10^−7^ m is located to the left of the larger sphere with radius *R*_2_ = 10^−6^ m (Figure 1). The interaction energy between the two spheres, 𝐸, is calculated by Equation (10) and plotted against the distance between the centers of the two spheres, (≥*R*_1_ + *R*_2_ = 1.5 × 10^−6^ m). (**A**) The ion concentration, *C*, of the electrolyte (and the respective Debye length from Equation (A2)) is: red curve: 0.007 mol/m^3^ (*λ_D_* = 1.15 × 10^−7^ m); blue curve: 0.01 mol/m^3^ (*λ_D_* = 9.62 × 10^−8^ m); and black curve: 0.013 mol/m^3^ (*λ_D_* = 8.44 × 10^−8^ m), and the temperature in the case of each curve is *T* = 300 K. (**B**) The ion concentration of the electrolyte is: green dotted curve: 0 mol/m^3^ (*λ_D_* = ∞ m); blue curve: 0.000001 mol/m^3^ (*λ_D_* = 9.62 × 10^−6^ m); red curve: 0.00001 mol/m^3^ (*λ_D_* = 3.04 × 10^−6^ m); and black curve: 0.0001 mol/m^3^ (*λ_D_* = 9.62 × 10^−7^ m), and the temperature in the case of each curve is *T* = 300 K. (**C**) The system’s temperature (and the respective Debye length) is: blue curve: 340 K (*λ_D_* = 1.02 × 10^−7^ m); red dotted curve: 310 K (*λ_D_* = 9.78 × 10^−8^ m); and black curve: 280 K (*λ_D_* = 9.30 × 10^−8^ m), and the electrolyte’s ion concentration in the case of each curve is 0.01 mol/m^3^. In the case of our calculations, the surface-charge density of each charged sphere is *ρ*_*s*_ = −0.266 × *C*/m^2^.

**Figure 3:**
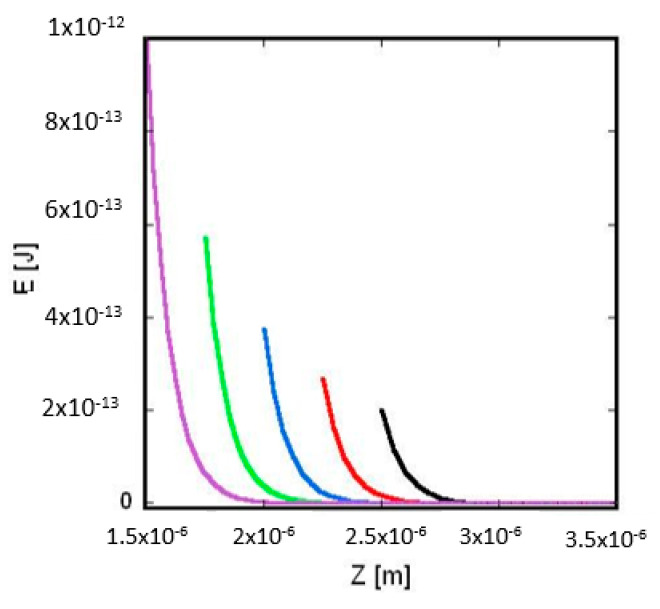
*Interaction energy of two charged spheres surrounded by electrolyte (dependence from radius):* the interaction energy between the two spheres, 𝐸, is plotted against the distance between the centers of the two spheres, *Z*. The total charge of the left and right sphere is *Q*_1_ = −8.3566 × 10^−13^ *C* and *Q*_2_ = −3.34265 × 10^−12^ *C*, respectively. The radius of the right sphere (see Figure 1) is *R*_2_ = 10^−6^ m, the electrolyte’s ion concentration is *C* = 0.01 mol/m^3^, the temperature is *T* = 300 K and the respective Debye length (calculated by Equation (A2)) is *λ_D_* = 9.62 × 10^−8^ m. Purple curve: *R*_1_ = 5 × 10^−7^ m; green curve: *R*_1_ = 7.5 × 10^−7^ m; blue curve: *R*_1_ = 1 × 10^−6^ m; red curve: *R*_1_ = 1.25 × 10^−6^ m; black curve: *R*_1_ = 1.5 × 10^−6^ m.

In Figure 2 and Figure 3, based on Equation (10), the interaction energy between two charged spheres (surrounded inside and outside by electrolyte) are calculated as a function of the distance between the centers of the spheres.

## 4. Discussion

Here, by using the recently generalized shell theorem [1], Equation (10) is derived to calculate the electric interaction energy between two charged spheres surrounded by electrolyte. Because of the increased screening effect of the electrolyte’s ions (i.e., with decreasing Debye length), at any given *Z* distance between the spheres, the interaction energy decreases with increasing electrolyte ion concentration (Figure 2A,B). The primary reason of this decrease is that the last factor of Equation (10) $\left(e^{-Z/\lambda_{D}}\right)$, at a given *Z* decreases fast when the Debye length, *λ_D_*_,_ decreases because of the increasing electrolyte ion concentration (see Figure 2A,B). On the other hand, *λ_D_* increases with increasing temperature (see interaction energy between two charged spheres (see Figure 2C)).

By increasing the radius *R*_1_, the 𝐸 vs. *Z* curves are shifting to the right (see Figure 3) because the lowest value of Z_𝑚i𝑛_ (= *R*_1_ + *R*_2_) increases. In addition, at Z_𝑚i𝑛_ the electric interaction energy *E*(Z_𝑚i𝑛_) is getting smaller. This is the case because with increasing *R*_1_ the distance between the charges of the spheres is increasing and, thus, the screening effect of the electrolyte’s ions increases too.

By using Equation (10), one can calculate the electric interaction energy between two charged spheres surrounded inside and outside by electrolyte. This equation is a generalization of the Coulomb equation (for charge–charge interaction in a vacuum [4]). One can get from Equation (10) an equation by taking the infinite long Debye length (that is the case at zero electrolyte ion concentration, i.e., when $C\to 0\left[\frac{\mathrm{mol}}{m^{3}}\right]$ (see Equation (A7))):

(11) $$E\left(Z\right)=\frac{k_{e}Q_{1}Q_{2}}{\varepsilon_{r}Z}\left\{\underset{\lambda_{D}\to \infty }{\lim}e^{-Z/\lambda_{D}}\frac{\lambda_{D}}{R_{1}}sinh\left(\frac{R_{1}}{\lambda_{D}}\right)\frac{\lambda_{D}}{R_{2}}sinh\left(\frac{R_{2}}{\lambda_{D}}\right)\right\}=\;\frac{k_{e}Q_{1}Q_{2}}{\varepsilon_{r}Z}\left\{\underset{\lambda_{D}\to \infty }{\lim}e^{-Z/\lambda_{D}}\frac{\lambda_{D}}{R_{1}}\left[\frac{R_{1}}{\lambda_{D}}+\frac{1}{3!}{\left(\frac{R_{1}}{\lambda_{D}}\right)}^{3}+\frac{1}{5!}{\left(\frac{R_{1}}{\lambda_{D}}\right)}^{5}+\cdots \right]\right\}\left\{\underset{\lambda_{D}\to \infty }{\lim}\frac{\lambda_{D}}{R_{2}}\left[\frac{R_{2}}{\lambda_{D}}+\frac{1}{3!}{\left(\frac{R_{2}}{\lambda_{D}}\right)}^{3}+\cdots \right]\right\}=\frac{k_{e}Q_{1}Q_{2}}{\varepsilon_{r}Z}$$

Equation (11) is similar to the Coulomb equation except that $\varepsilon_{r}$ is not the relative permittivity of vacuum but the relative permittivity of the pure water. Calculating the curves in Figure 2 and Figure 3, constant electrolyte permittivity ($\varepsilon_{r}\;$= 78) was taken that is characteristic to pure water at a temperature of 300 K. Note that the relative permittivity of electrolytes depends on the temperature, ion concentration and type of the ions (see Appendix A). With increasing temperature and ion concentration, the relative permittivity of the electrolyte slightly and close to linearly decreases and affects the calculated value of the interaction energy too (see Appendix A).

By using Equation (10), one can also calculate the total electric interaction energy of several separated, charged spheres surrounded inside and outside by electrolyte Equation (12):

(12) $$=\sum_{i=1}^{N}\sum_{\begin{array}{c} j=1\\ j\neq i \end{array}}^{N}\frac{k_{e}Q_{i}Q_{j}{\lambda_{D}}^{2}}{\varepsilon_{r}R_{i}R_{j}Z_{ij}}sinh\left(\frac{R_{i}}{\lambda_{D}}\right)sinh\left(\frac{R_{j}}{\lambda_{D}}\right)e^{-Z_{ij}/\lambda_{D}}$$

where *N* is the number of spheres, *Q_i_* and *R_i_* are the total charge and radius of the *i*-th sphere, respectively, and *Z_ij_* (where *Z_ij_* > *R_i_* + *R_j_*) is the distance between the centers of the *i*-th and *j*-th sphere.

## 5. Conclusions

By using the recently generalized version of Newton’s shell theorem [1], analytical equations are derived to calculate the electric interaction energy between two separated, charged spheres surrounded outside and inside by electrolyte. This electric interaction energy is calculated as a function of the electrolyte’s ion concentration, temperature, distance between the spheres and the size of the spheres. At the same distance, the absolute value of the interaction energy decreases with increasing electrolyte ion concentration and increases with increasing temperature. At zero electrolyte ion concentration, the derived analytical equation transforms into the Coulomb Equation Finally, the analytical equation is generalized to calculate the electric interaction energy of N separated, charged spheres surrounded by electrolyte.

## Data Availability

Not applicable.

## Appendix A

The Poisson equation is [11]:

(A1) $$\nabla^{2}V\left(\underline{r}\right)=-\frac{\rho \left(\underline{r}\right)}{\varepsilon_{0}\varepsilon_{r}}$$

where *ρ*(*r*) is the charge density, *ε*_0_ is the vacuum permittivity and *ε*_r_ is the relative static permittivity.

The solution of the above Poisson equation is [11]:

(A2) $$V\left(\underline{r}\right)=ʃʃʃd^{3}{\underline{r}}^{\prime }\frac{\rho \left({\underline{r}}^{\prime }\right)}{4\pi \varepsilon_{0}\varepsilon_{r}\left|\underline{r}-{\underline{r}}^{\prime }\right|}$$

In the case of the Poisson–Boltzmann equation [11,12]:

(A3) $$-\frac{\rho \left(\underline{r}\right)}{\varepsilon_{0}\varepsilon_{r}}=\sum_{i}\frac{z_{i}qn_{i}^{0}}{\varepsilon_{0}\varepsilon_{r}}e^{-\frac{z_{i}qV\left(\underline{r}\right)}{k_{B}T}}$$

where *q* is the elementary charge (positive or negative depending on the charge of the *i*-th type of ion), *z_i_* is the charge number of the *i*-th type of ion, *k_B_* is the Boltzmann constant, *T* is the absolute temperature, ρ(*r*) is the charge density of the ions in the electrolyte and *ε_r_* is the relative static permittivity of the electrolyte.

The screened Poisson equation is [9]:

(A4) $$\nabla^{2}V\left(\underline{r}\right)-{\lambda_{D}}^{-2}V\left(\underline{r}\right)=-\frac{\rho_{ex}\left(\underline{r}\right)}{\varepsilon_{0}\varepsilon_{r}}$$

where *ρ*_*ex*_(*r*) is the density of the external charge at position *r*, *ε*_0_ is the electric constant and *ε_r_* is the relative static permittivity of the electrolyte and *λ_D_* is the Debye length. Note that Equation (A4) is valid if the electrolyte itself is electrically neutral. The solution of this equation is [11], i.e., the potential is the superposition of the so-called screened Coulomb potential of the external charges:

(A5) $$V\left(\underline{r}\right)={ʃʃʃ}^{\;}d^{3}{\underline{r}}^{\prime }\frac{\rho_{ex}\left({\underline{r}}^{\prime }\right)}{4\pi \varepsilon_{0}\varepsilon_{r}}\frac{e^{-\left|\underline{r}-{\underline{r}}^{\prime }\right|/\lambda_{D}}}{\left|\underline{r}-{\underline{r}}^{\prime }\right|}$$

The Debye length in an electrolyte is calculated by [13]:

(A6) $$\lambda_{D}={\left(\frac{\varepsilon_{0}\varepsilon_{r}k_{B}T}{e^{2}N_{a}\sum_{j=1}^{N}c_{j}^{0}q_{j}^{2}}\right)}^{1/2}$$

where ε_0_ = 8.85 × 10^−12^ *C*^2^*J*^−1^m^−1^ is the vacuum permittivity, $\varepsilon_{r}$ is the relative static permittivity of the electrolyte, *k_B_* = 1.38 × 10^−23^ *JK*^−1^ is the Boltzmann constant, *T* is the absolute temperature, *e* = 1.6 × 10^−19^ *C* is the charge of a positive monovalent ion, *N*_𝑎_ = 6 × 10^23^ mol^−1^ is the Avogadro’s number, *C*_*j*_^0^ mol/m^3^ is the mean concentration of the *j*-th species of ions in the electrolyte and *q*_*j*_ is the number of elementary charges in an ion of the *j*-th species (e.g., in the case of bivalent ions *q*_*j*_ = 2). In this paper, we consider overall neutral electrolytes containing only monovalent positive and negative ions of the same concentration, *C*. In this case, Equation (A6) is simplified to:

(A7) $$\lambda_{D}={\left(\frac{\varepsilon_{0}\varepsilon_{r}k_{B}T}{e^{2}N_{a}2C}\right)}^{1/2}$$

The relative static permittivity of the neutral electrolyte $\left(\varepsilon_{r}\right)$ depends on (a) the type of ions solved in the water, (b) the temperature and (c) the concentration of the ions (C). The temperature dependence of $\varepsilon_{r}$ for pure water (from 0 °C to 100 °C) is [14]:

(A8) $$\varepsilon_{r}\left(t,C\right)=87.74-\left[0.40008\times t\right]+\left[9.398\times 10^{-4}\times t^{2}\right]-\left[1.41\times 10^{-6}\times t^{3}\right]$$

where t is the temperature in Celsius and $C=0\left[\frac{mol}{m^{3}}\right]$ is the ion concentration in the pure water. The dependence of the static permittivity from the NaCl concentration $\left(C\left[\frac{mol}{m^{3}}\right]\right)$ of the electrolyte (at temperature t=20 °C) is [15]:

(A9) $$\varepsilon_{r}\left(t,C\right)=\varepsilon_{r}\left(t,0\right)\times \left[1-\alpha C\right]$$

where $\alpha =6.6\times 10^{-3}\left[\frac{m^{3}}{mol}\right]$ and $\varepsilon_{r}\left(t,0\right)=81$. Calculating the curves in Figure 2 and Figure 3, a constant relative permittivity of the electrolyte ($\varepsilon_{r}=78$) was considered. This is the relative permittivity of pure water at 300 K. By means of Equations (A8) and (A9), one may take into consideration the temperature and ion-concentration dependence of the relative permittivity of the electrolyte when calculating the interaction energy between two charged spheres by Equation (10). Since the relative permittivity slightly and close to linearly decreases, with increasing temperature and ion concentration one can estimate how the interaction energy is changing. Based on Equation (A8), if the temperature of pure water is higher than 300 K the relative permittivity is lower than 78 and the interaction energy, calculated by Equation (10) becomes higher than the interaction energy calculated with constant relative permittivity ($\;\varepsilon_{r}=78$). If, beside the higher temperature, the ion concentration of the electrolyte is $C>0\left[\frac{mol}{m^{3}}\right],$ the relative permittivity is even lower than 78 and, thus, the interaction energy calculated by Equation (10) becomes even higher than the interaction energy calculated with constant relative permittivity ($\;\varepsilon_{r}=78$).
